# Aquaporin 1, a potential therapeutic target for migraine with aura

**DOI:** 10.1186/1744-8069-6-68

**Published:** 2010-10-25

**Authors:** Guang-Yin Xu, Fen Wang, Xinghong Jiang, Jin Tao

**Affiliations:** 1 Key Laboratory of Pain Research & Therapy, Department of Neurobiology and Institute of Neuroscience, Soochow University, Suzhou 215123, P.R. China; 2 Department of Internal Medicine, University of Texas Medical Branch, TX 77555, USA

## Abstract

The pathophysiology of migraine remains largely unknown. However, evidence regarding the molecules participating in the pathophysiology of migraine has been accumulating. Water channel proteins, known as aquaporins (AQPs), notably AQP-1 and AQP-4, appears to be involved in the pathophysiology of several neurological diseases. This review outlines newly emerging evidence indicating that AQP-1 plays an important role in pain signal transduction and migraine and could therefore serve as a potential therapeutic target for these diseases.

## Introduction

Migraine is a chronic, paroxysmal, neurovascular disorder that can start at any age, and affects up to 6% of males and 18% of females in the general population [1]. Two major forms of migraine exist: migraine without aura and migraine with aura. An often debilitating, unilateral, throbbing headache typically characterizes attacks of migraine without aura. This type of headache which may last 4 to 72 hours is aggravated by physical activity, and is accompanied by autonomic symptoms such as vomiting, nausea, photophobia, and phonophobia [2]. However, the attack may also be preceded by premonitory symptoms (prodrome) in some patients. In one third of migraineurs, the headache phase is preceded or accompanied by transient focal symptoms of neurologic aura (migraine with aura). These are usually visual but may also involve sensory disturbances, speech difficulties, and motor symptoms [1].

**Figure 1:**
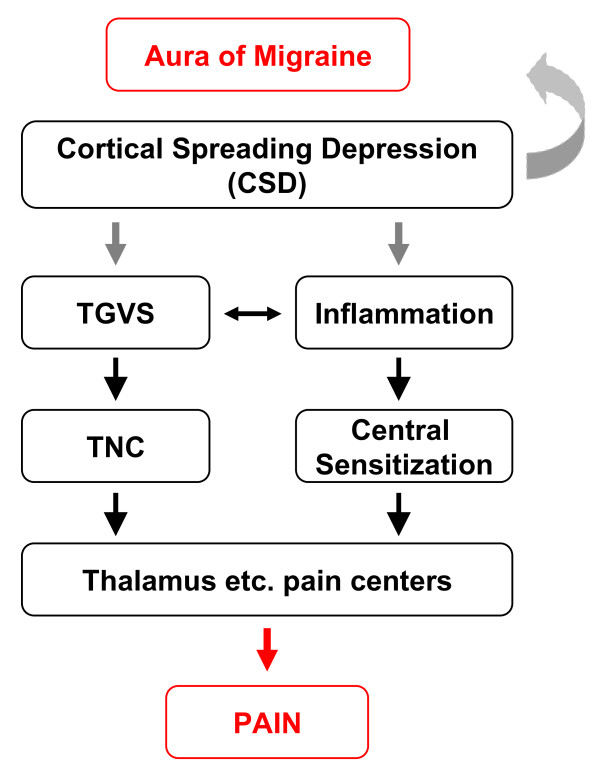
**Hypothesized model of nociceptive pathways in migraine pathophysiology**. Cortical spreading depression (CSD) is associated with the release of neurotransmitters and metabolites, which are thought to be the underlying cause of the migraine aura. These neurotransmitters may also activate perivascular trigeminal nerve endings, resulting in activation of the trigeminovascular system (TGVS), and subsequently, the trigeminal nucleus caudalis (TNC), which conveys signals to the thalamus en route to the somatosensory cortex. In addition, activation of the TGVS induces meningeal neurogenic inflammation, which could result in the central sensitization.

Much progress has now been made in elucidating the mechanisms underlying the aura and headache phases of migraine attacks [3]. The migraine aura is thought to be caused by "cortical spreading depression" (CSD), a wave of intense neuronal activity that slowly progresses over the cortex and is followed by a period of neuronal inactivity. Elevated extracellular levels of potassium and glutamate might be crucial for the initiation and propagation of CSD. During the headache phase, activation of the trigeminovascular system (TGVS) plays a crucial role. The TGVS consists of the meningeal and superficial cortical blood vessels that are innervated by the trigeminal nerve, which projects into the trigeminal nucleus caudalis in the brainstem, which in turn, projects to higher-order pain centers (Figure 1). Evidence from animal experiments suggests that CSD might activate the TGVS, potentially linking the mechanisms for aura and headache [3]. Although the mechanisms of the aura and headache are relative well understood, hardly is anything known about how migraine attacks are initiated. Such knowledge is important to design effective, well-tolerated and prophylactic treatments. Genetic factors may have the possibility to play an important role in migraine by lowering the trigger threshold for migraine attacks. Genetic research in the field of migraines has mainly focused on the identification of genes involved in familial hemiplegic migraine (FHM), a rare monogenic subtype of migraine with aura. The main clinical reason for this validity is that the symptoms of aura and headache are similar, apart from the hemiparesis associated with FHM, and that most patients with FHM also have attacks of common migraine [2]. Three genes for FHM have been identified: CACNA1A (FHM1), encoding the pore-forming α1-subunit of voltage-gated neuronal Cav2.1 (P/Q-type) calcium channels, ATP1A2 (FHM2), encoding the α2-subunit of glial cell sodium-potassium (Na^+^-K^+^) pumps, and SCN1A (FHM3), encoding the pore-forming α1-subunit of voltage-gated neuronal Nav1.1 sodium channels [3-5]. With the identification of these genes, it seems that the FHM- and likely other common types of migraine- are disorders of disturbed ion transport, or ionopathies, has gained increasing acceptance. However, direct convincing evidence that the CACNA1A, ATP1A2, or SCN1A gene is involved in common forms of migraine is largely lacking. Moreover, the precise mechanism by which these mutations cause FHM is unknown currently. It is also not clear whether the mutations represent a gain or loss of function. It is, therefore, to identify new potential therapeutic targets to gain insight into the triggering mechanisms of migraine attacks.

Aquaporins (AQPs), a family of water channel proteins, became a very hot area of research in biochemistry and molecular cell biology, with increasing physiological, medical, and biotechnological implications. The characteristics, tissue distribution, functions and some pathophysiological implications of individual AQPs are briefly presented below and references of detailed reviews on each topic are given. The recent advances on relating AQPs and the pathophysiology of migraine are the focus of this review.

### Structure, distribution and functions of AQPs

**Figure 2:**
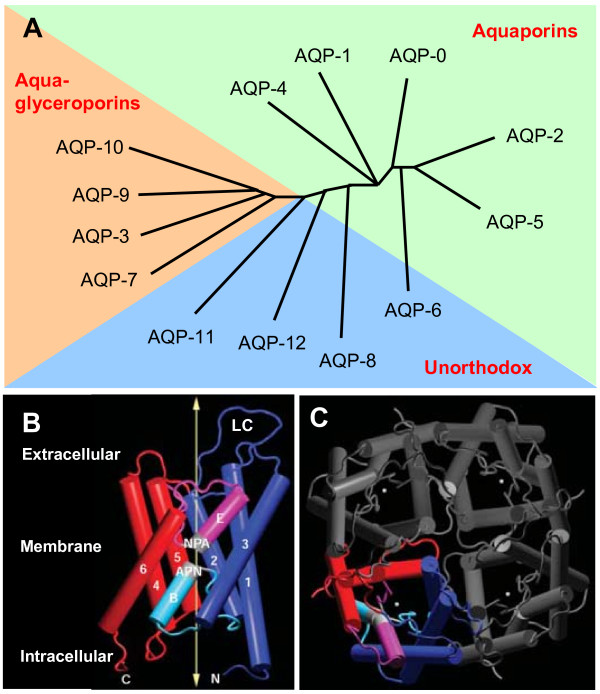
**The aquaporin family tree and the structure of AQP-1**. **A**., This phylogenetic tree shows the relationship of the thirteen water channel proteins in mammals (AQP-0-AQP-12). The assignments in the phylogenetic tree roughly correlate with permeability characteristics. The aquaporins are generally permeated only by water. The aquaglyceroporins are permeated by water and small solutes such as glycerol. The unorthodox were recently named as AQP-11 and AQP-12 were previously named superaquaporins or subcellular AQPs and AQP-6 and AQP-8 were previously named aquaporins. **B, **The monomeric structure of the aquaporin-1 (AQP-1) is shown with membrane-spanning helices numbered 1-6 displayed as rods and the location of the membrane and the long extracellular C loop (LC). The aminoterminal half of the molecule is shown in purple and light blue, and the carboxy-terminal half is shown in red and pink. Loops B and E, which fold into the membrane to form the pore, are labeled, as are the conserved NPA motifs (shown in light grey). Portions of loops B and E form α-helices, and are therefore shown as rods. The arrow highlights the route taken by water, which can move in both directions through the channel. C, carboxyl terminus; N, amino terminus. **C**, The AQP-1 tetramer, as seen from above. Asterisks denote the location of the water pore in each subunit. Modified from King after the original published in ref. (15) and reproduced with permission from Nature Publishing Group.

The aquaporins are small hydrophobic membrane proteins. They selectively transport water and some small solutes across plasma membranes in mammals, plants, and lower organisms [6]. To date, 13 different subtypes of mammalian AQPs (AQP-0-12) have been identified with differing tissue distributions that appear related to their functional roles in water-transporting [7-10]. Most authors consider that out of these, seven are aquaporins (AQP-0, AQP-1, AQP-2, AQP-4, AQP-5, AQP-6, and AQP-8), four are aquaglyceroporins (AQP-3, AQP-7, AQP-9, and AQP-10), whereas AQP-11 and AQP-12 are "superaquaporins" or subcellular AQPs since they have unusual NPA (Asn-Pro-Ala) motifs [11,12]. AQP-11 is a 271-amino-acid protein in which the second NPA motif is conserved but the first motif is substituted by NPC (Asn-Pro-Cys) in both mice and humans [13]. AQP-12 is a 290- or 295-amino-acid aquaporin that is closely related to AQP-8 in humans and to AQP-0 and AQP-6 in mice [14]. The first NPA motif in AQP-12 is substituted by an NPT (Asn-Pro-Thr) motif in both species. Recently, AQP-6, AQP-8, AQP-11, and AQP-12 were named "unorthodox" AQPs (Figure 2A) [15], whose functions are currently being elucidated. The majority of studies would characterize AQP-6 as a unique, Hg^2+^- and low-pH-activated, multipermeable channel. Mouse AQP-8 increases urea but not glycerol permeability, whereas human AQP-8 is neither urea nor glycerol permeable [13]. In contrast, there is still no evidence for significant transport of water, glycerol, urea, or ions, contradicting a recent report that found significant water permeability of AQP-11 when AQP-11 was reconstituted into liposomes [16]. Furthermore, the significance of AQP-12 is completely unknown, and AQP-12 has not been characterized functionally [13]. There is a considerable body of information about AQP structure from electron and x-ray crystallography and molecular dynamics simulations [11,17,18], demonstrating AQP monomers (~30 kDa) containing six membrane-spanning helical domains surrounding a narrow aqueous pore (Figure 2B). The helices of each AQP-1 monomer that is positioned on the outside face of the tetramer are hydrophobic, whereas those that are placed towards the centre of the tetramer are hydrophilic. AQP monomers are super-assembled in membranes as tetramers (Figure 2C), which are similar to many ion channel family members, such as potassium channels and cyclic nucleotide-gated (CNG) channels [19,20]. Unlike ion channels, however, the channel for water permeability does not reside at the fourfold axis (the centre of the tetramer). Instead, each monomer contains a channel (Figure 2C).

AQPs are expressed in various tissues including urinary, respiratory, digestive, and nervous systems [21], and they provide the molecular basis for water transport in the tissues. AQP channels facilitate bi-directional water transport across the plasma membrane in response to osmotic gradients created by solute movement. Water permeation through AQP channels is characterized by sensitivity to mercurial agents like HgCl_2 _[21,22] and tetraethylammonium (TEA) [23]. Most of AQPs (except for AQP-4 and AQP-7) are sensitive to HgCl_2_. The genetic manipulation of rodents and the identification of humans with altered aquaporin genes have provided considerable insights into aquaporin-related physiology. For example, the AQP-1 or AQP-4 deficient mice have impairments in urinary concentrating ability [24,25], cerebral fluid balance [26], corneal fluid balance [27], hearing [28], and water transport in the lungs [29,30]. Defective secretion in salivary and submucosal glands [31,32] and altered lung fluid transport [31] have been shown in AQP-5 knockout mice. Defects in AQP-2 have been shown to be linked to nephrogenic diabetes insipidus [33,34]. These examples demonstrate that AQPs are required for normal water homeostasis, and are likely to be important players in a variety of human diseases.

### AQPs in the central nerve system (CNS)

AQPs in the CNS appear to be of great physiological and pathological importance, especially given the rigid physical constraint that is imposed by the bony cranium. However, current knowledge of aquaporin expression and function in the nervous system is very limited. Generally, AQPs are involved in water movement in nervous tissue; nevertheless, recent data would suggest the involvement of AQPs in neurotransmission. Several studies have reported the expression of AQP water channels, AQP-1, AQP-4, and AQP-9 in the brain [9,35,36], and AQP-4 and AQP-9 in the spinal cord [37]. AQP-4, the predominant water channel in the central nervous system, is mainly expressed in astrocytes throughout the brain and spinal cord [38]. AQP-4 plays a role in cerebral edema, glial cell migration and neuroexcitation [39]. However, mechanisms of AQP-4 modulation of cortical spreading depression (CSD) remain unclear. AQP-9 is found in a subset of astrocyte processes that form the glia limitans [36] and specialized ependymal cells in the brain and spinal cord [37].

Brain AQP-1 is mainly expressed in the cerebrospinal fluid (CSF)-facing membranes of the ventricular choroid plexus, where it regulates the formation of CSF presumably due to its functions as a water pore and ion channel [40]. Interestingly, various neuropathological conditions involve the up-regulation of AQP-1 expression in the CNS. Upregulation of brain AQP-1 expression is found in Alzheimer patients [41], Creutzfeldt-Jakob disease [42], traumatic brain injury patients, and human hemangioblastomas. In all cases, the elevated AQP-1 expression seems to occur in astrocytes residing in diseased brain tissue, even though these astrocytes and other glial cells do not normally express AQP-1. The possible roles of increased expression of AQP-1 in the etiology of different neuropathological conditions are still unknown, but it is likely that AQP-1 up-regulation may contribute to edema and cyst formation-typical outcomes in most of these pathological conditions. Unfortunately, the mechanisms that control AQP-1 gene expression in the CNS, under normal or pathological conditions, are still poorly understood. However, Kim *et al *[43] showed that the thyroid transcription factor-1 up-regulates AQP-1 synthesis and thus facilitates CSF formation in the brain. Moreover, in CNS, hypertonicity induces AQP-1 synthesis via extracellular signal-regulated kinases, p38 kinase, and c-Jun terminal kinase [44].

In the spinal cord, AQP-1 is also expressed in the ependymal cells lining the central canal, but more robustly in the sensory fibers of the superficial laminae of the dorsal horn [45-47]. Considering the expression of AQP-1 in the dorsal horn, it has been suggested that AQP-1 has a role in physiological pain sensation, known as nociception. Two groups [48,49] showed recently that AQP-1-deficient mice are less sensitive to noxious thermal stimuli or capsaicin (the pungent component of chili peppers which also activates nociceptive skin afferents), and even in human dorsal horn with neuropathic pain [50]. However, Shields et al. [46] provided evidence against this role. They reported that AQP-1-null mice did not have altered nociception. The possibility for relating AQP-1 and nociception at spinal cord level, remains speculative.

### AQPs in peripheral sensitization of nociception

A growing body of evidence showed that AQPs appear to be involved in the peripheral sensitization of nociceptors. AQP-1 is expressed in small afferent sensory nerve fibers in the peripheral nervous system. In research published by Oshio's group, both the small neurons and small afferent nerve fibers in normal mouse dorsal root ganglion were shown the co-localization of AQP-1 and capsaicin receptor (TRPV1) [51]. The TRPV1 receptors have the function of transmitting the painful stimuli *in vivo *[52]. Furthermore, recent studies showed that thermal inflammatory pain perception was greatly reduced in AQP1^-/- ^mice evoked by bradykinin, prostaglandin E2, and capsaicin as well as reduced cold pain perception [53]. Nav1.8 currents and expression were significantly reduced in AQP1^-/- ^mice DRG neurons. The reduction in Nav1.8 currents would contribute to the impairment in repetitive AP firing and to the accelerated adaptation observed in AQP-1-deficient DRG neurons. In addition, immunoprecipitation studies and single molecule tracking indicated a physical interaction between AQP-1 and Nav1.8. These data provide a physical and functional link between AQP-1 expression and Nav1.8 function, and implicate the involvement of AQP1 in DRG neurons for the perception of inflammatory thermal pain [53]. Interestingly, GFAP-positive, injured Schwann cells in the peripheral nervous system (PNS) are known to express AQP-1 [54], but not AQP-4, a protein abundant in spinal cord astrocytes [55]. AQP-2 expression was not detectable either in the spinal cord or in the dorsal root ganglia of naive rats. However, AQP-2 expression was dramatically increased in small-diameter dorsal root ganglia neurons in response to chronic constriction injury treatment [56]. These data support the hypothesis that AQP-1 and AQP-2 might be involved in pain processing under inflammatory and neuropathic nerve injury conditions. However, the detailed mechanisms of such roles warrants to be further investigated.

### AQP-1 in migraine pathophysiology

The major symptom of migraine, the headache pain, is mediated by neuronal activity along the trigeminovascular pathway. Activation and sensitization of primary afferent neurons (PANs) in the trigeminal ganglion (TG) is the first step in driving this nociceptive pathway. Using immunofluorescent staining, Shields et al. have described that AQP-1 is heavily expressed in a population of small diameter primary sensory neurons of TG, and co-localized with a marker of peptidergic nociceptors, substance P [46]. In addition to the dorsal root ganglion, the expression of TRPV1 in rat TG was detected recently [57]. Furthermore, the co-localization of 5-HT_1B _receptors with substance P was also identified in the human TG [58].

**Figure 3:**
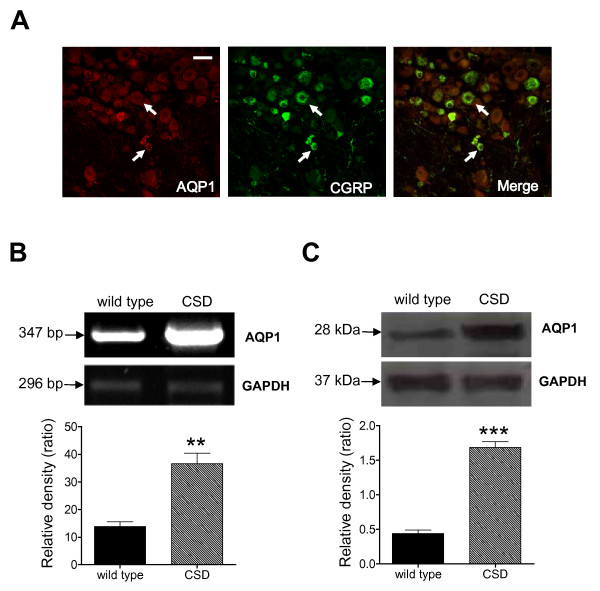
**The expression of AQP-1**. **A**., Co-localization of AQP-1 and CGRP in mouse TG neurons. Merge of double labeling of AQP-1 positive (left, red) and CGRP positive (middle, green) were shown in yellow (right). Bar = 50 μm. **B**, AQP-1 mRNA was detected in mouse upper cervical and medullary dorsal horn under control and CSD conditions. CSD dramatically upregulated AQP-1 mRNA expression (n = 3, **P < 0.01 *vs*. wild type). **C**, AQP-1 protein expression was detection in mouse TNC under control and CSD conditions. CSD greatly enhanced the AQP-1 protein expression in mouse TNC (n = 4, ***P < 0.001 *vs*. wild type).

**Figure 4:**
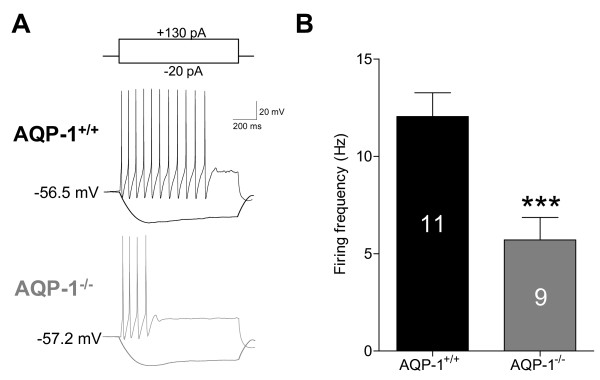
**Decrease in neuronal excitability in AQP-1 knockout mice**. AQP-1 deficient significantly decreased the firing frequency of upper cervical dorsal horn neurons evoked by superthreshold current stimulation. Numbers of the cells recorded were shown in columns. ***P < 0.001 *vs*. AQP-1^+/+^.

Based on these observations, it is tempting to speculate that AQP-1 might be co-expressed with TRPV1 receptors, substance P, or 5-HT_1B _receptors in the neuronal cells and small diameter afferent nerve fibers of trigeminal ganglion and their connecting structures, thus involved in the pathophysiology of migraine. This hypothesis is highly supported by our recent results that AQP-1 is co-expressed with calcitonin gene related peptide (CGRP), a nociceptive marker, in TG neurons (Figure 3A). Given that migraine represents a chronic neurological disease, we hypothesized that in a model of migraine AQP-1 expression would be elevated. Indeed, in wild type mice, epidural KCl evoked repetitive CSDs induced up-regulation of AQP-1 expression at both mRNA (Figure 3B) and protein levels (Figure 3C) in upper cervical dorsal horn, a key component in the migraine pathway. The AQP-1 expression remained unaltered in TG in a mice model of perioral acute inflammatory pain although the AQP-2 expression was remarkably increased in small-sized neurons and Schwann cells [56]. The intracellular redistribution of AQP-2 was also observed in this animal model. These data support the idea that AQP-1 upregulation in TG might be disease specific. To further determine the role of AQP-1, we found that the deficiency of AQP-1 (mice kindly provided by Dr. Alan Verkman from University of California, San Francisco) significantly decreased the firing frequency of upper cervical dorsal horn neurons (Figure 4). In addition, silencing the mouse AQP-1 gene *in vivo *using a viral RNA interference approach abolished the elevated avoidance to light of migraine model, which models the photophobia often observed in patients with migraine (data not shown). These observations point to the hypothesis that AQP-1 may serve as a potential therapeutic target for migraine [59].

## Conclusions

Migraine is among the more debilitating diseases, and current treatment modalities are unsatisfactory in more than half of the patients [3]. More specific, well-tolerated, and effective methods of prophylaxis are desired. AQPs represent just one of many exciting potential therapeutic targets. Other possible candidates include NOTCH3, the causative gene for CADASIL (cerebral autosomal dominant arteriopathy with subcortical infarcts and leukoencephalopathy), and SLC1A3, encoding the EAAT1 (excitatory amino acid transporter and glutamate transporter [3]. The development of genetically sensitized mouse models has more or less opened up a completely new field for migraine research. Whereas previous research concentrated on elucidating the mechanisms of CSD and intracranial nociception, newer candidates will facilitate research into increased sensitivity to migraine triggers and metabolic homeostasis. In addition to treatment of acute attacks, a better understanding of the mechanism of migraine attack triggers will help in the development of specific preventive therapies.

## Competing interests

The authors declare that they have no competing interests.

## Authors' contributions

All authors have read and approved the final manuscript. JT performed the electrophysiological recording, prepared the figures and drafted the manuscript. XHJ participated in the design of the study and helped to draft the manuscript. FW performed the molecular biology experiments and helped to analyze the data. GYX conceived of the study, coordinated the project, helped to interpret the data, and helped to draft the manuscript.
